# Preferred Advanced Airway Device Use Among Adults With Out-of-Hospital Cardiac Arrest

**DOI:** 10.1001/jamanetworkopen.2025.2913

**Published:** 2025-04-02

**Authors:** Christopher B. Gage, Henry E. Wang, Jacob C. Kamholz, Jonathan R. Powell, Ashish R. Panchal

**Affiliations:** 1 National Registry of Emergency Medical Technicians, Columbus, Ohio; 2 Department of Emergency Medicine, The Ohio State University Wexner Medical Center, Columbus; 3 The Ohio State University, College of Public Health, Columbus.

## Abstract

This cross-sectional study assesses the temporal trends in advanced airway strategies for US adults with out-of-hospital cardiac arrest from 2018 to 2023.

## Introduction

Airway management for out-of-hospital cardiac arrest (OHCA) is a core part of cardiopulmonary resuscitation (CPR) and recent technological innovations and procedural refinements have allowed for various advanced airway strategies in the US.^1^ A study using a subset of electronic health records from the National Emergency Medical Services (EMS)^2^ found advanced airway strategies in adult patients with OHCA shifted over the past 10 years, with the proportion of supraglottic airway (SGA) attempts surpassing endotracheal intubation (ETI) attempts since 2020.^3^ Using a larger dataset from the National EMS Information System (NEMSIS), we sought to determine whether this finding represented national-level practice.

## Methods

In this cross-sectional study, we leveraged data from the NEMSIS, which includes 96% of US EMS agencies, to evaluate differences in advanced airway methods used by EMS personnel for adults with OHCA between 2018 and 2023. Patients were included when cardiopulmonary resuscitation (CPR) was documented (NEMSIS variable e.Arrest.01: presence of cardiac arrest before and after EMS arrival). Differences in advanced airway choice and patient population are presented as frequencies (%) and medians (IQRs) calculated using Stata/ME 18 (StataCorp LLC). As a sensitivity analysis of patient selection,^3^ we compared airway attempt strategy proportions with a broader case definition including cases when CPR or defibrillation was documented across multiple variables (NEMIS variable eProcedures.03 and others) (Supplement 1). This approach aimed to account for inconsistencies in self-reported documentation (eg, cases marked as no CPR in eArrest.01 but with CPR procedures recorded elsewhere). The American Institutes for Research International Review Board deemed the study exempt due to use of deidentified data. We followed the STROBE reporting guideline.

## Results

Using the focused case definition, we identified 2 201 170 patients with OHCA from 2018 to 2023. Median (range) patient age was 66 (52-77) years and 1 376 958 (61.8%) were male (Table). A total of 765 865 patients (34.8%) had 1 or more advanced airway attempts. ETI was the predominant choice for advanced airways throughout the study period (82.3% in 2018 and 69.2% in 2023) compared with to SGA (26.5% in 2018 and 40.1% in 2023) (Figure). While the broad case selection used in the sensitivity analysis included more patients than the focused case selection (broad: 2 365 295; focused: 2 201 170; difference, 7.2%), we found similar airway attempt strategy proportions between the focused and broad case selection. A significant change in airway management occurred between 2019 and 2020; the proportion of ETI use decreased from 81.4% to 68.9% and the proportion of SGA use increased from 27.5% to 39.2%. This change may be attributable to airway policy changes due to COVID-19 in 2020.

**Table.** Characteristics of Populations With Out-of-Hospital Cardiac Arrest in the National Emergency Medical Services Information System Included in the Focused Case Selection

Characteristic	No. (%)					
	2018 (n = 174 967)	2019 (n = 267 948)	2020 (n = 419 229)	2021 (n = 443 011)	2022 (n = 456 121)	2023 (n = 439 894)
Age, median (IQR)	66 (53-77)	66 (53-77)	66 (53-77)	65 (52-77)	66 (52-77)	66 (52-77)
Gender						
Male	108 632 (62.1)	167 310 (62.4)	260 871 (62.2)	277 395 (62.6)	285 356 (62.6)	277 394 (63.1)
Female	65 327 (37.3)	99 264 (37.0)	156 849 (37.4)	163 431 (36.9)	168 648 (37.0)	160 235 (36.4)
Missing data	1008 (0.6)	1374 (0.5)	1509 (0.4)	2185 (0.5)	2117 (0.5)	2265 (0.5)
Urbanicity^a^						
Urban	140 317 (80.2)	217 818 (81.3)	346 950 (82.8)	364 346 (82.2)	381 462 (83.6)	369 569 (84.0)
Rural	14 031 (8.0)	20 610 (7.7)	28 905 (6.9)	31 703 (7.2)	30 709 (6.7)	37 056 (8.4)
Suburban	14 053 (8.0)	20 937 (7.8)	31 232 (7.4)	34 225 (7.7)	34 236 (7.5)	25 025 (5.7)
Missing data	6566 (3.8)	8583 (3.2)	12 142 (2.9)	12 737 (2.9)	9714 (2.1)	8244 (1.9)

^a^ For 2022, percentages may not sum to 100 due to rounding.

**Figure.**
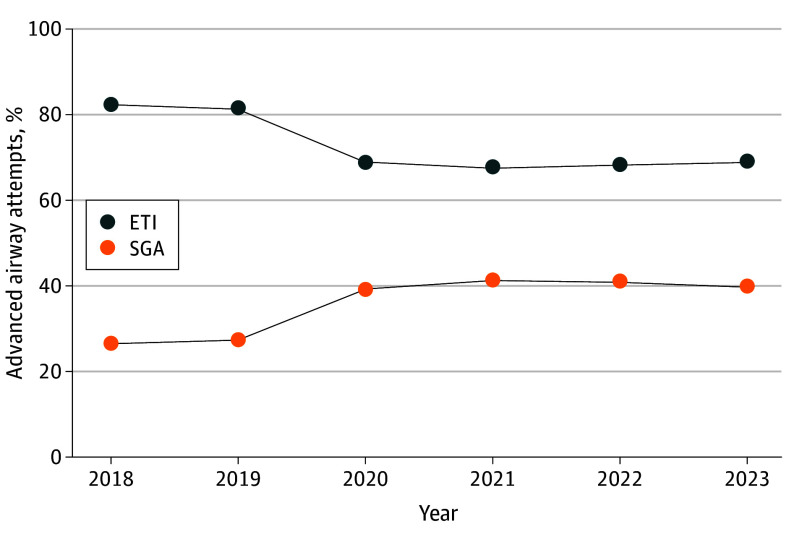
Advanced Airway Attempts With Endotracheal Intubation (ETI) and Supraglottic Airway (SGA) Devices Among Patients Who Required Advanced Airways Figure shows the proportion (displayed as percentages for clarity) of advanced airway attempts using an ETI or SGA among the subset of patients with out-of-hospital cardiac arrest included in the focused case selection method. Annual percentages may be greater than 100% since patients may have received more than 1 airway device or multiple attempts.

## Discussion

This cross-sectional study found that ETI remains the predominant advanced airway strategy in adults with OHCA across the US. Airway strategy patterns from 2020 to 2023 indicate stable choices by EMS clinicians, with no substantial shift favoring SGA. This consistency in ETI preference suggests that current trends in airway management in patients with OHCA rely on the need to establish a definitive airway despite mixed evidence regarding airway device outcomes.^4^

These findings contrast with a prior study^3^ using a single-source EHR dataset that suggested a rise in SGA use over ETI. Potential differences between the prior study and current study could stem from biases, including represented EMS agencies and selection of patients with OHCA.^3^ The NEMSIS dataset offers a more comprehensive representation of national EMS practices than other datasets.^2,5,6^ Given that agency type and regional practices vary across the country, the broader NEMSIS dataset likely provides a more accurate reflection of national OHCA practices than a single EHR system can achieve. We found no differences between airway strategy proportions among focused or broad case selections, making this potential bias minimal. One study limitation is that all data are self-reported. Additionally, although we noted increases in the number of patients with OHCA over time, there were no substantial demographic changes between years, suggesting that the changes seen in airway strategy proportions are not a product of population changes. Our analysis revealed that ETI remains the predominant airway choice in adults with OHCA, with no evidence of SGA superseding ETI.

## Notes

Supplement 1. **eMethods.** Variables found in focused and broad case selection methods

Supplement 2.
Data Sharing Statement
