# Supplementary material for: Preferred Advanced Airway Device Use Among Adults With Out-of-Hospital Cardiac Arrest
Source: JAMA Netw Open. 2025 Apr 2;8(4):e252913. doi: 10.1001/jamanetworkopen.2025.2913 (PMC11966300; doi:10.1001/jamanetworkopen.2025.2913)
Supplement: Supplement 1. — eMethods. Variables found in focused and broad case selection methods [file jamanetwopen-e252913-s001.pdf]

Supplemental Online Content

Gage CB, Wang H, Kamholz JC, Powell JR, Panchal, AR. Preferred advanced airway device use among adults with out-of-hospital cardiac arrest. *JAMA Netw Open*. 2025;8(4):e252913. doi: 10.1001/jamanetworkopen.2025.2913

eMethods. Variables found in focused and broad case selection methods

This supplemental material has been provided by the authors to give readers additional information about their work.

© 2025 Gage CB et al. *JAMA Network Open*.

eMethods. Variables found in focused and broad case selection methods.

Method	Variable	Code	Description
**Focused**	eArrest_01 - Presence of Cardiac Arrest Before and After EMS Arrival	3001003	Yes, Prior to EMS Arrival
		3001005	Yes, After EMS Arrival
**Broad**	eArrest_01 - Presence of Cardiac Arrest Before and After EMS Arrival	3001003	Yes, Prior to EMS Arrival
		3001005	Yes, After EMS Arrival
	eArrest_03 – Resuscitation Attempted By EMS	3003001	Attempted Defibrillation
		3003005	Initiated Chest Compressions
	eArrest_09 – Type of CPR Provided	3009001	Compressions-Continuous
		3009003	Compressions-External Band Type Device
		3009005	Compressions-External Plunger Type Device
		3009007	Compressions-External Thumper Type Device
		3009009	Compressions-Intermittent with Ventilation
		3009011	Compressions-Other Device
	eArrest_07 – AED Use Prior to EMS Arrival	3007005	Yes, With Defibrillation

© 2025 Gage CB et al. *JAMA Network Open*.
