# Supplementary material for: Preferred Advanced Airway Device Use Among Adults With Out-of-Hospital Cardiac Arrest
Source: JAMA Netw Open. 2025 Apr 2;8(4):e252913. doi: 10.1001/jamanetworkopen.2025.2913 (PMC11966300; doi:10.1001/jamanetworkopen.2025.2913)
Supplement: Supplement 2. — Data Sharing Statement [file jamanetwopen-e252913-s002.pdf]

Data Sharing Statement

Gage. Preferred Advanced Airway Device Use Among Adults With Out-of-Hospital Cardiac Arrest. *JAMA Netw Open*. Published April 02, 2025. doi:10.1001/jamanetworkopen.2025.2913

Data
Data available: Yes
Data types: Data (not involving human participants)
How to access data: All data can be requested through the NEMSIS TAC as public use data.
When available: With publication

Supporting Documents
Document types: None

Additional Information
Who can access the data: Anyone whose proposed use of the data has been approved by the NEMSIS TAC.
Types of analyses: Contact the NEMSIS TAC for more information (https://nemsis.org/using-ems-data/request-research-data/)
Mechanisms of data availability: Contact the NEMSIS TAC for more information (https://nemsis.org/using-ems-data/request-research-data/)
